# Does cardiac rehabilitation favour the young over the old?

**DOI:** 10.1136/openhrt-2016-000450

**Published:** 2016-08-03

**Authors:** Abdulrahman Al Quait, Patrick Doherty

**Affiliations:** Department of Health Sciences, Faculty of Science, University of York, Heslington, UK

**Keywords:** AGE GROUP

## Abstract

**Background:**

Although cardiac rehabilitation (CR) is a proven intervention in reducing cardiovascular mortality and morbidity there is concern that CR programme delivery may not yield comparable outcomes across age groups.

**Purpose:**

This study sought to determine if the outcomes achieved after completing CR were influenced by age in patients with coronary heart disease.

**Method:**

Patients were stratified into 2 age groups: young (18–65 years) and elderly (>65 years). Pre-CR and post-CR assessments were used to compute changes in 9 CR outcomes (body mass index (BMI), waist size, hyperlipidaemia, hypertension, smoking, walking fitness, physical activity, anxiety and depression). Pearson's χ^2^ test was used to examine the association between the age groups and outcome. Data was extracted from the UK National Audit from July 2010 to June 2015.

**Results:**

A total of 203 012 young patients (55.1±7.9 years, 78% male) and 262 813 elderly patients (76.1±6.9 years, 63.9% male) were analysed. Young patients had a better ratio of improvement across a wide range of risk factors in particular smoking cessation (OR=3.3, p<0.001) while elderly patients had a better ratio of improvement in body shape risk factors BMI (OR=1.3, p<0.001), waist size in women (OR=1.3, p=0.016).

**Conclusions:**

Age is a significant predictor of outcomes following CR. While elderly patients achieve better outcomes in body shape risk factors, younger patients clearly achieve better outcomes across a wider range of risk factors in particular smoking cessation.

**Key questions**

**What is already known about this subject?**

- Recent data indicate that cardiac rehabilitation outcomes vary by age group and it has been shown that a single approach to cardiac rehabilitation programme delivery may not fit all patient age groups.

**What does this study add?**

- This study will contribute to our understanding of the factors that determine the outcomes and inform the future cardiac rehabilitation offer.

**How might this impact on clinical practice?**

- Cardiac rehabilitation programmes should take account of the impact of age by tailoring the intervention to ensure that all age groups gain the most from their cardiac rehabilitation experience.

## Introduction

Cardiac rehabilitation (CR) programmes are designed to provide a range of lifestyle and medical interventions to reduce cardiovascular mortality and morbidity through the promotion of a healthy lifestyle, psychosocial well-being and subsequent reduction in risk factors. A wealth of evidence-based studies has proven the benefits of CR for patients.

Anderson *et al* performed a Cochrane systematic review where 63 studies were analysed, randomising 14 486 patients with coronary heart disease (CHD) to CR or usual care. The results showed that CR reduced cardiovascular mortality in 12 month follow-up studies (relative risk (RR) 0.74 (95% CI 0.64 to 0.86)) and hospital admissions (RR 0.82 (95% CI 0.70 to 0.96)) in the shorter term (<12 months follow-up) with no significant effect on total mortality, myocardial infarction (MI) and revascularisation. The predominant group in the review population, however, was middle-aged male patients (median age 56 years, 49.3–71) with women representing <15% of the total population.^1^ Although the benefits of CR are well established, most of this research data comes from middle-aged and older patients.^2^

The overall ageing of the population in the UK,^3^ in addition to the improvement of survival rates in patients with cardiovascular diseases (CVD), has created a large number of adults older than 65 years who are eligible for CR.^4^ In 1988, the prevalence of CVD in men aged over 65 years was around 23.5% while in 2011 it rose to 31.3%.^5^ The ageing population of eligible CR patients with multiple morbidity, increased frailty and disability presents challenges to CR delivery that are not matched by innovation in current CR programmes. Despite improvement in CR uptake in recent years, it has been shown that the average age of medically managed post MI patients, for example, starting a CR programme is 8.5 years below the age of patients that enter cardiology services.^6^

The British Association for Cardiovascular Prevention and Rehabilitation (BACPR) is responsible for producing national guidelines for CVD prevention and rehabilitation, which aim to improve the safety and standards of CR programmes throughout the UK.^7^ The National Audit of Cardiac Rehabilitation (NACR), which is funded by the British Heart Foundation (BHF), is responsible for collecting clinical data from CR programmes before and after attending rehabilitation which is then published as annual reports.^8^

There has been little quantitative analysis of the association between CR outcomes and different age groups and this paper attempts to show that one size CR programme may not fit all patient groups. Using the NACR data, this study sought to investigate the extent by which age determined the outcome following CR. This study will contribute to our understanding of the factors that determine the outcomes and potentially inform the CR offer.

## Methods

### Data collection

This is an observational retrospective study where data was retrieved for individual patients with CHD from the UK NACR covering the period 1 July 2010 to 30 June 2015. The NACR has the approval to collect anonymised patient data for a range of clinical variables and to use this data to improve the quality of services and patients outcomes.^9^ The collected data pertains to patients who undergo CR in the UK and includes details of the patients' demographics, clinical conditions and lifestyles. Information is entered manually into the NACR through a secure online portal or uploaded to the Health and Social Care Information Centre (HSCIC). Data is gathered by clinicians and by specifically designed questionnaires.^10^ The NACR seeks annual approval from HSCIC to use the collected data to monitor and report on the quality of CR in the UK.^8^ NACR also has Section 251 exemption which means that patient consent is not sought as the data is anonymised by the HSCIC before reaching the NACR team.

Patients were stratified into two predefined age groups where the retirement age in the UK was used as a cut point: young (aged 18–65 years) and elderly (aged >65 years). Patients were included in the analyses if they had completed CR and had pre-CR and post-CR assessments. Pre-CR and post-CR assessments were used to compute the change in nine modifiable risk factors (body mass index (BMI), waist size, hyperlipidaemia, hypertension, smoking, fitness, physical activity, anxiety and depression).

### Statistical analysis

The analyses were conducted on SPSS 23. Significance levels were set at the 5% level using Pearson's χ^2^ test to examine the association between the two age groups and any outcome changes experienced. To compute the difference in the outcome achieved following an individual CR programme, the record of pre-CR assessment was subtracted from the record of post-CR assessment. Since the outcomes in the NACR data were measured in either a continuous scale or Likert/dichotomous scale variables, two methods to compute the outcomes were used. To compare the relative odds of the occurrence of the outcome of interest, the OR was calculated.^11^

The outcomes measured in continuous scale variables include: total cholesterol, systolic blood pressure, BMI, waist size and fitness measured by Incremental Shuttle Walking Test (ISWT). To measure the change in total cholesterol levels, patients who had measurements more than (5 mmol/L) pre-CR assessment and measurements below (5 mmol/L) post-CR assessment were classified as improved in a computed categorical variable named ‘total cholesterol change’. Patients who did not demonstrate this change were classified in the no change group.

For systolic blood pressure outcomes and BMI a similar method was used, with 140 mm Hg and 30 kg/m² used as cut points respectively. Since the meaningful change in waist size is based on respondent gender, this variable was stratified by male and female subgroups with 102 cm waist size in men and 88 cm waist size in women.

Outcomes measured in Likert/dichotomous scale variables include smoking, physical activity level, anxiety level and depression level. For the dichotomous variables of smoking and physical activity, improvement was recorded if patient responses changed positively between pre-CR and post-CR. The other variables are all Likert scale variables and the outcome computation was conducted by computing the difference variable with a simple subtraction method. From that, change statistics were generated for all variables.

## Results

The first set of analyses examined the impact of age on CR outcomes. Only patients with pre-CR and post-CR assessments were included in the outcome analysis. A total of 203 012 young patients (55.1±7.9 years, 78% male) and 262 813 elderly patients (76.1±6.9 years, 63.9% male) were analysed. The baseline characteristics of the study population is summarised in table 1.

**Table 1:** Baseline characteristics of both groups

Factor	Young group (<65 years)	Elderly group (>65 years)	Difference significance	Effect size*
N	203 012	262 813	<0.001	r=0.01
Mean age (SD) (years)	55.1 (7.9)	76.1 (6.9)	<0.001	r=0.83
Male (%)	78%	63.9%	<0.001	V=0.15
Ethnicity (British) (%)	82.5%	89.1%	<0.001	V=0.11
Employment (FT) (%)†	49.7%	6.3%	<0.001	V=0.70
Obesity (BMI>30 kg/m^2^) (%)	29.3%	18.9%	<0.001	V=0.12
Male waist circumference (%)‡	37.5%	37.3%	0.60	V=0.00
Female waist circumference (%)§	65.8%	59.1%	<0.001	V=0.07
Smokers (%)	38%	14.4%	<0.001	V=0.27
Comorbidities (+3) (%)	15.8%	22.7%	<0.001	V=0.09
Total cholesterol (+4) (%)¶	73.7%	60.7%	<0.001	V=0.14
Clinically anxious (%)	19%	9.6%	<0.001	V=0.17
Clinically depressed (%)	10.3%	5.9%	<0.001	V=0.09
Hypertensive (%)**	72.6%	64.6%	<0.001	V=0.09
Fitness level (SD)††	402.1 m (166.2)	279.4 m (136.5)	<0.001	r=0.14
Moderate physical activity (%)‡‡	24.7%	20.3%	<0.001	V=0.05
Vigorous physical activity (%)§§	6.2%	2.9%	<0.001	V=0.08
Outpatient CR duration (SD)¶¶	59.5 (46.6)	61.5 (46.5)	0.001	r=0.02

*r=Pearson correlation, V=Cramer's V effect sizes.

†Ratio of patients in either FT or PT jobs.

‡Waist size >102 cm.

§Waist size >88 cm.

¶Ratio of patients with total cholesterol more than 4 mmol/L.

**Ratio of hypertensive patients (blood pressure over 140 mm Hg systolic and 90 mm Hg diastolic).

††Average ISWT metres walked in each group.

‡‡Ratio of patients taking moderate physical activity for 150 min/week.

§§Ratio of patients taking vigorous physical activity for 75 min/week.

¶¶Outpatient CR mean duration in days.

BMI, body mass index; CR, cardiac rehabilitation; Employment FT, full time; ISWT, Incremental Shuttle Walk Test.

At baseline, the populations of the two groups were significantly different in the following categories: BMI (effect size 0.12), waist size for females (effect size 0.07), smokers (effect size 0.27), ethnicity (effect size 0.11), employment status (effect size 0.70), total cholesterol (effect size 0.14), anxiety (effect size 0.17), depression (effect size 0.09), hypertension (effect size 0.09) fitness level (effect size 0.14), moderate physical activity (effect size 0.05) and vigorous physical activity (effect size 0.80).

**Figure 1:**
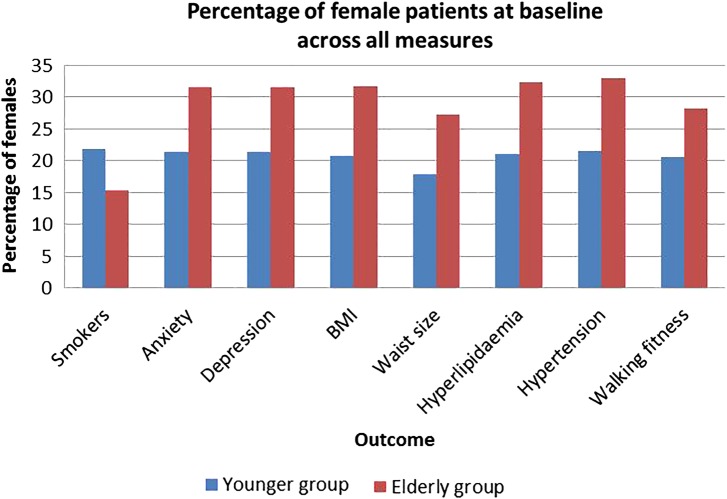
Percentage of female patients at baseline for each measure. BMI, body mass index.

Figure 1 presents the breakdown by female gender according to the measured variables at baseline. It is apparent from this chart that the ratio of females at baseline is higher in the elderly group except for smokers (21.9% in young vs 15.4% in elderly).

From the data presented in table 2, a positive correlation was found between older age and change in body shape risk factors based on ORs with the exception of male waist size which was statistically not significant (p=0.145). On the other hand, younger patients achieved better outcomes in all other risk factors as shown in table 3. The most striking result to emerge from the data analysis is that young patients are about three times more likely to quit smoking than elderly patients (OR 3.3).

**Table 2:** Outcomes where elderly patients performed better than young patients

Risk factor	N		p Value	OR
BMI	Young	9027	<0.001	1.3
	Elderly	6740		
Waist size (female patients)	Young	1569	0.016	1.3
	Elderly	2189		

BMI, body mass index.

**Table 3:** Outcomes where young patients performed better than elderly patients

Risk factor	N		p Value	OR
Total cholesterol	Young	5392	<0.001	1.3
	Elderly	3407		
Blood pressure	Young	6308	<0.001	1.4
	Elderly	9727		
Smoking	Young	18 351	<0.001	3.3
	Elderly	18 550		
ISWT	Young	2972	<0.001	1.7
	Elderly	2765		
Moderate physical activity 150 min/week	Young	37 581	<0.001	1.1
	Elderly	39 556		
Vigorous physical activity 75 min/week	Young	35 611	<0.001	1.4
	Elderly	37 549		
Anxiety	Young	28 940	<0.001	1.7
	Elderly	31 435		
Depression	Young	28 883	<0.001	1.3
	Elderly	31 405		

ISWT, Incremental Shuttle Walk Test.

## Discussion

This study aimed to assess the importance of age as a predictor for CR outcomes. Elderly patients are usually frail and at higher risk of complications following a cardiac event compared with their younger counterparts.^12^ These complications may lead to longer hospital stays and greater vulnerability to subsequent clinical deconditioning.^13^ Moreover, elderly patients have higher rates of impairment in their physical activity after a cardiac event.^14^ Those differences should reflect on the achieved CR outcomes, especially if we consider that CR programmes are based on studies with middle-aged patients.^12^ ^15^ In this study, we have shown that the elderly patients are the least fit cohort with a higher number of comorbidities than their younger counterparts.

The difference in sample number between the two age groups could be associated with the increased prevalence of CVD in older patients^12^ and it has been reported that being younger in age is a predicting factor of CR drop-out.^16^ We can also conclude that the CR referral rates in the UK are not significantly affected by older age, as has been reported in many studies.^17–22^ Table 1 shows that older women are more likely to complete a CR programme compared with younger women (22% in younger cohort and 36.1% in the elderly). Other demographic differences between the two populations included ethnicity and employment. In both groups the majority of patients were British (82% for the young and 89.1% for the elderly). Although the difference in ethnicity between the two groups is statistically significant (p<0.001) this could be explained by the large sample sizes and should not have a major impact on the outcome results (effect size 0.11).

As the retirement age in the UK is 65 years, it is likely that fewer patients attending CR will have full time or part time jobs which this study confirmed (6.3% compared with 49.7% in the younger group). Theoretically being retired allows people more time to attend CR sessions and engage with the desired lifestyle changes. However, the elderly group did less well on most of the other outcomes which makes this explanation alone insufficient. Other differences possibly affecting the outcomes were the core CR duration and the time between the baseline and post CR assessments. In both groups these times were found to be similar; assessment period 84.4 days (51.6) and 87.3 days (52.9), CR duration 59.5 days (46.6) and 61.5 days (46.5) for the young and elderly groups respectively.

The prevalence of being overweight or obese is well documented in cardiac patients. These characteristics are considered independent risk factors for CHD.^23^ ^24^ At baseline, obese patients were more prevalent in the younger cohort (BMI>30 kg/m^2^=36.2%) while prevalence of large waist circumference was similar in both groups (≈58%, p=0.73, V<0.001). Improvement in weight was best observed in the elderly group (OR 1.3). Waist size reduction, however, was most notable in female elderly patients (OR=1.3) while older males improved by only 10% (OR 1.1).

A strong relationship was evident between the younger aged group and improvements in the other CR outcomes. The current study found that total cholesterol and systolic blood pressure were more reduced in the younger cohort than the elderly by 30% and 40%, respectively. Although the contribution of CR towards improvement in hyperlipidaemia and hypertension is well documented,^25^ the effect of medical drugs cannot be excluded.

The ISWT distance (metres) is routinely used as a measure of exercise capacity (fitness) in the CR population. In 2014, Houchen-Wolloff *et al* established the minimum clinically important difference in ISWT following CR. In their study, they tested 220 patients, mean (SD) age was 65 years (10.5), BMI 28.4 kg/m^2^ (5.1), 170 male. The ISWT mean change was 65.2 m (95% CI 55.4 to 74.9 m) after CR (p<0.001) suggesting that the minimum clinically important difference for the ISWT following CR is 70 m (95% CI 51.5 to 88.5 m)m.^26^

In this study, the younger group had a better fitness level and physical activity status at baseline (table 1). These differences at baseline can be attributed to the fact that there is a gradual deterioration in muscle mass, muscle strength and oxygen uptake usually associated with ageing.^26^ Despite this, there is a growing body of literature which indicates that regular exercise among older people regardless of their degree of frailty, with or without underlying chronic disease, may absorb the consequences of ageing on exercise capacity.^27^ In addition, it has been reported that individuals with low baseline levels may demonstrate the largest overall gain in fitness and physical activity due to the law of initial values which has also been shown in CR patients.^27^ However, this study, using clinical data, has been unable to demonstrate that as the younger group acquired better walking fitness and physical activity status post CR than the elderly. These results are in agreement with Sandercock *et al*'s^27^ meta-analysis which showed significantly larger gains in fitness in the youngest age group (<55 years). The other factors that may contribute to the capacity to gain in fitness include exercise frequency intensity and dose, in addition to patient characteristics such as gender and health condition. There is, therefore, a definite need for baseline fitness testing to tailor the exercise prescription for each individual patient that takes account of age.^7^ ^28^

CR programmes should strive to support patients who have decided to cease smoking.^6^ Overall, randomised control trials have found a significant increase in smoking cessation among those randomised to CR.^29^ The results of this study indicate that the ratio of individuals who achieved smoking cessation between the two groups was statistically significant (p<0.001, OR 3.3) and in favour of the younger cohort. Since the ratio of elderly patients who quit smoking post-CR is trivial (≈1.2%) and given that cigarette smoking was identified as a CR participation barrier,^30^ further studies should be conducted to evaluate the benefits of enrolling elderly patients in smoking cessation programmes.

Substantial evidence indicates that psychological distress is a significant risk factor for cardiac diseases and adversely affects recovery after major cardiac events.^2^ Significant reductions in anxiety (OR=1.7) and depression (OR=1.3) levels were achieved in the younger group. Although previous research found that anxiety levels could improve by 32% in patients >70 years post a comprehensive CR programme,^12^ this analysis shows that the proportion of improvement in anxiety and depression is inversely correlated with age.

## Conclusion

The characteristics of patients at the point of entry to CR can vary significantly according to patient's age which is an important consideration when tailoring an intervention for patients. While elderly patients achieve better outcomes in body shape risk factors, younger patients achieve much better outcomes across a wider range of risk factors in particular with regards to smoking cessation. Current CR programmes should be underpinned by a baseline assessment and fully implement a tailored intervention to ensure that all age groups gain the most from their CR experience.
